# Rapid culture‐independent loop‐mediated isothermal amplification detection of antimicrobial resistance markers from environmental water samples

**DOI:** 10.1111/1751-7915.14227

**Published:** 2023-02-03

**Authors:** Marwa M. Hassan, Arnoud H. M. van Vliet, Owen Higgins, Liam P. Burke, Alexandra Chueiri, Louise O'Connor, Dearbháile Morris, Terry J. Smith, Roberto M. La Ragione

**Affiliations:** ^1^ Department of Comparative Biomedical Sciences, School of Veterinary Medicine, Faculty of Health and Medical Sciences University of Surrey Guildford UK; ^2^ Molecular Diagnostics Research Group, School of Biological and Chemical Sciences University of Galway Galway Ireland; ^3^ Antimicrobial Resistance and Microbial Ecology Group, School of Medicine University of Galway Galway Ireland; ^4^ Centre for One Health, Ryan Institute University of Galway Galway Ireland; ^5^ School of Biosciences, Faculty of Health and Medical Sciences University of Surrey Guildford UK

## Abstract

Environmental water is considered one of the main vehicles for the transmission of antimicrobial resistance (AMR), posing an increasing threat to humans and animals health. Continuous efforts are being made to eliminate AMR; however, the detection of AMR pathogens from water samples often requires at least one culture step, which is time‐consuming and can limit sensitivity. In this study, we employed comparative genomics to identify the prevalence of AMR genes within among: *Escherichia coli*, *Klebsiella*, *Salmonella enterica* and *Acinetobacter*, using publicly available genomes. The *mcr‐1*, *blaKPC* (KPC‐1 to KPC‐4 alleles), *blaOXA‐48*, *blaOXA‐23* and *blaVIM* (VIM‐1 and VIM‐2 alleles) genes are of great medical and veterinary significance, thus were selected as targets for the development of isothermal loop‐mediated amplification (LAMP) detection assays. We also developed a rapid and sensitive sample preparation method for an integrated culture‐independent LAMP‐based detection from water samples. The developed assays successfully detected the five AMR gene markers from pond water within 1 h and were 100% sensitive and specific with a detection limit of 0.0625 μg/mL and 10 cfu/mL for genomic DNA and spiked bacterial cells, respectively. The integrated detection can be easily implemented in resource‐limited areas to enhance One Health AMR surveillances and improve diagnostics.

## Graphical abstract

A schematic illustration of the culture‐independent preparation method for water samples and detection of five AMR targets. The 8‐tube strip contains different AMR LAMP primers targeting as follows: (1) *mcr‐1*, (2) *blaKPC*, (3) *blaOXA‐48*, (4) *blaOXA‐23*, (5) *blaVIM*, (6) positive control 1, (7) positive control 2 and (8) negative control, with a pink colour indicating a negative result, and a yellow colour indicating a positive result. The figure was created by M.M.H. using Biorender.com in March 2021 and edited in June 2022.

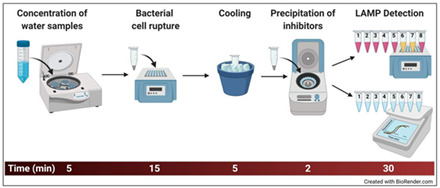

## INTRODUCTION

In 2019, it was estimated that approximately 5 million deaths were associated with bacterial AMR, of which 1.27 million were directly due to bacterial AMR (Murray et al., 2022). It is currently estimated that the mortality rate from infections caused by antimicrobial‐resistant bacteria is approximately 700,000 deaths per year, and this figure is projected to increase to >10 million by 2050 (O'Neill, 2014). Additionally, approximately 60% of the global emerging infectious diseases are of zoonotic origins (Jones et al., 2008; Zhongming & Wei, 2020), with bacterial infections having a higher prevalence worldwide (Cantas & Suer, 2014; Jones et al., 2008; Rabozzi et al., 2012; Zhongming & Wei, 2020). Therefore, the rapid increase in AMR is imposing a higher risk on public health every day (Cantas & Suer, 2014; Tacconelli et al., 2018; Zhongming & Wei, 2020). One of the major antibiotic resistance classes is extended spectrum beta‐lactamase (ESBL), where the production of beta‐lactam degrading enzymes is the most frequently reported antibiotic resistant mechanism in Gram‐negative bacteria (Bush & Jacoby, 2010; Tacconelli et al., 2018). They are classified structurally into four molecular classes: classes A, B, C and D, where class B ESBLs are metalloenzymes that require zinc to facilitate hydrolysis, and functionally into three groups: group 1 cephalosporinases (molecular class C), group 2 serine beta‐lactamases (molecular classes A and D) and group 3 metallo‐beta‐lactamases (molecular class B) such as *blaVIM* (group 3a) (Bush & Jacoby, 2010). Functional group 2 is one of the largest beta‐lactamases groups including carbapenemases such as *blaOXA‐23* (group 2df), *blaOXA‐48* (group 2df) and *blaKPC* (group 2f) (Bush & Jacoby, 2010). ESBLs, particularly carbapenemases, have been reported worldwide in medical (Flokas et al., 2017; Tacconelli et al., 2018), veterinary (Salgado‐Caxito et al., 2021) and environmental settings (Pormohammad et al., 2019). Carbapenemases are particularly challenging due to the limited therapeutic choices. Therefore, ESBL and carbapenem‐resistant Enterobacteriaceae, carbapenem‐resistant *Acinetobacter baumannii* and carbapenem‐resistant *Pseudomonas aeruginosa* are of the top critical priority of World Health Organisation's (WHO) priority pathogens list (Tacconelli et al., 2018). Furthermore, ESBLs are frequently encoded on plasmids and other mobile genetic elements that can carry other antibiotic resistances leading to the increased spread of multi‐drug resistances, which further limits treatment choices (Flokas et al., 2017). Thus, colistin, a polymyxin antibiotic, is considered one of the last resort antibiotics for treating carbapenem resistant and multidrug resistant Gram‐negative infections (Cherak et al., 2021) and is one of the highest critically important antimicrobials (World Health Organization, 2019). However, since the discovery of mobile colistin resistance (*mcr‐1*), several *mcr* genes have been reported among Gram‐negative isolates worldwide (Hussein et al., 2021), highlighting the importance of continued monitoring and AMR surveillance for *mcr*.

Contaminated and poor‐quality water sources represent a significant risk for the spread of AMR genes (Hiller et al., 2019; Huijbers et al., 2015; Larsson & Flach, 2022). Several AMR markers such as *ampC*, *tet*, *sul*, *ermB*, *vanA* and *bla* genes have been detected in effluents from wastewater treatment plants and surface water, leading to increased chances of horizontal gene transfer of antibiotic resistance in the environment (Farrell et al., 2021; Hiller et al., 2019). Thus, different mitigation strategies have been developed to treat and remove antibiotic resistant bacteria and AMR genes from contaminated water (Farrell et al., 2021; Hiller et al., 2019). However, standard detection of coliforms from water samples requires a membrane filtration step followed by at least one culturing step. The culturing step is advantageous in enriching the number of bacterial cells and increasing sensitivity, but can limit the number of species identified and prevent the detection of viable but non‐culturable bacteria. DNA extraction can also be performed directly from filtered water samples, but the detection efficiency is highly dependent on the extraction method, treatment protocols, bacterial load and the abundance of AMR genes (Hiller et al., 2019).

Rapid and sensitive diagnostics are crucial to aid in the understanding of the spread of zoonotic pathogens and to assist in control and prevention (Bird & Mazet, 2018), especially for pathogens harbouring highly prevalent AMR genes such as ESBLs and carbapenemases. Culture‐based diagnostics are time consuming and of variable sensitivity due to the nature of the culture enrichment. They often necessitate confirmatory molecular detection or even another culture‐based AMR detection, which directly affects sensitivity (Kurupati et al., 2004; Trotter et al., 2019). Therefore, over the last decade, there have been significant developments in the field of diagnostics and molecular detection, to rapidly and sensitively detect zoonotic pathogens and associated AMR (Bird & Mazet, 2018). However, it still remains a challenge to develop diagnostics for resource‐limited settings that require limited or no expertise. These diagnostic assays are particularly essential to be implemented in low‐ and middle‐income countries and remote locations, where the lack of AMR surveillance and control measures is evidenced (Bird & Mazet, 2018; Rabozzi et al., 2012). Loop‐mediated isothermal amplification (LAMP) is a rapid and low‐cost detection method that can be used in resource‐limited settings as once developed and validated it requires limited equipment and very limited to no expertise to perform (Hassan et al., 2022). Loop‐mediated isothermal amplification was first developed in 2000 by Notomi et al. (2000). Since 2000, it has been widely used for detecting different pathogens such as *Staphylococcus* (Diribe et al., 2014), *Shigella*, *E. coli* (Song et al., 2005) and SARS‐CoV‐2 (Rohaim et al., 2020), in addition to the detection of several AMR targets such as *blaOXA‐48* (Sasano et al., 2021), *blaCTX‐M‐9* (Thirapanmethee et al., 2014) and other ESBL genes (Feng et al., 2021).

In this study, we utilised publicly available genome sequences and repositories to study the prevalence of beta‐lactamase and *mcr* genes in *E. coli*, *Klebsiella*, *Salmonella enterica* and *Acinetobacter*. Five AMR markers of medical and veterinary relevance and contribute to the WHO pathogens priority list were targeted as follows: plasmid‐mediated colistin resistance (*mcr‐1*), *Klebsiella pneumoniae* carbapenem resistance (KPC), oxacillin‐hydrolysing β‐lactamases (OXA‐48/ 48‐like variants and OXA‐23) and Verona integron‐encoded metallo‐β‐lactamase (VIM) were selected for the development and validation of a rapid AMR LAMP detection platform. The developed AMR LAMP assays utilised fluorescence and colorimetric detection for rapid direct testing. The five AMR LAMP assays were then integrated with a rapid and culture‐independent sample preparation method to facilitate direct detection from water samples. We demonstrated the successful culture‐independent detection of AMR targets in spiked tap and pond water samples in less than 1 h with a detection limit of 10 cfu/mL.

## METHODS

### Comparative genomics analysis

Data sets of *E. coli*, *Klebsiella*, *Salmonella enterica* and *Acinetobacter* were accessed during April–May 2020 at the NCBI Pathogens database (https://www.ncbi.nlm.nih.gov/pathogens/isolates/#) and downloaded as comma‐separated value (CSV) files. The field with antibiotic resistance genes were distributed over individual columns, and only *bla* (beta‐lactamase) and *mcr* genes (colistin resistance) were enumerated, including the individual allele number. Data sets were manually transferred to new worksheets for further processing, with the aim of selecting the most common alleles in each of the four target organisms (*E. coli*, *Klebsiella*, *Salmonella* and *Acinetobacter*). The genomes positive for these alleles were then selected for further investigation to check for inclusion of geographic regions. The AMRfinder database (https://github.com/ncbi/amr/wiki/AMRFinder‐database) was used to extract specific *bla* and *mcr* allele DNA sequences for comparison and primer design. The datasets, metadata and the number of sequences used are detailed in (Van Vliet et al., 2020) (Tables S1 and S4).

### LAMP primer design for AMR targets

Different LAMP primers were designed for the detection of the selected AMR markers: *mcr‐1*, *blaKPC*, *blaOXA‐48*, *blaOXA‐23* and *blaVIM*. The target AMR gene sequences and their corresponding alleles were selected based on the high prevalence and incidence from the comparative genomics study (Van Vliet et al., 2020), and downloaded from NCBI database for multiple sequence alignment using MEGA X software (Kumar et al., 2018). Conserved sequences were used for designing LAMP primers with LAMP designer 1.15 software (Optigene) and PrimerExplorer V4 and V5 (Eiken Chemical Co. LTD.). Three pairs of LAMP primers: forward and reverse of external (F3 and B3), internal (FIP and BIP) and loop primers (LF and LB) were designed for each target gene taking into considerations product length (130–200 bp (Notomi et al., 2000)) and annealing temperatures to ensure high LAMP amplification efficiency. The validated AMR LAMP primers and the length of designed target sequences are listed in Table 1.

**TABLE 1:** LAMP primers sequences of selected AMR targets and their corresponding amplicon size.

AMR target	Primers sequences (5′‐ > 3′) (mers)^a^		Length of target sequence (bp)
mcr‐1	F3	TCATGTCAGCTTCAATGGCT (20)	149
	B3	GATCCAGCGTATCCAGCAC (19)	
	FIP	GCCGCACGATGTGACATTGCGCGCGATACTTTCCCACA (38)	
	BIP	CGACGGCGTATTCTGTGCCGGGTATTTGGCGGTATCGACA (40)	
	LF	GCCATCGATCTTGGCAAGC (19)	
	LB	TGTATGTTCAGCTATCTGGGCGC (23)	
KPC	F3	GTGTACGCGATGGATACC (18)	171
	B3	GTCATGCCTGTTGTCAGATAT (21)	
	FIP	CAGCGGCAGCAAGAAAGCCAGGCGCAACTGTAAGTTA (37)	
	BIP	GCTTGCTGGACACACCCATTTCCGAGATGGGTGACC (36)	
	LF	CTTGAATGAGCTGCACAGTG (20)	
	LB	TCCGTTACGGCAAAAATGC (19)	
OXA‐48	F3	ATATCGCCACTTGGAATCG (19)	176
	B3	TTGACAATACGCTGGCTG (18)	
	FIP	TGCTCATACGTGCCTCGCCGATGAAATATTCAGTTGTGCC (40)	
	BIP	ATGAGGACATTTCGGGCAATGTAAAGCTGATTTGCTCCGT (40)	
	LF	TTTGGCGGGCAAATTCTTG (19)	
	LB	GGCTCGACGGTGGTATTC (18)	
OXA‐23	F3	ATCGGATTGGAGAACCAGA (19)	166
	B3	CCTCTTGAATAGGCGTAACC (20)	
	FIP	GTTCCTGATAGACTGGGACTGCAAGGTCATTTACCGCTTGG (41)	
	BIP	GCGACGTATCGGTCTTGATCTCATCAACCTGCTGTCCAATT (41)	
	LF	GCTTCTCCTAGTGTCATGTCTT (22)	
	LB	CGTATTGGTTTCGGTAATGCTG (22)	
VIM	F3	CACGCATTCTCTAGAAGGAC (20)	174
	B3	GAATGACGACCTCTGCTTC (19)	
	FIP	ACCGTATAGCACGTTCGCTGTAGAGCTCTTCTATCCTGGTG (41)	
	BIP	GTTGTCAAGCACGTCTGCGTTTGAATCCGCTCAACGG (37)	
	LF	TACAACCAGATTGTCGGTCG (20)	
	LB	GATGCCGATCTGGCTGAA (18)	

^a^ F3 and B3 are the forward and reverse external primers; FIP and BIP are the forward and reverse inner primers; LF and LB are the forward and reverse loop primers, respectively.

### Bacterial strains and culture conditions

Bacterial strains were sourced from the National Collection of Type Cultures (NCTC), rehydrated and cultured according to the supplier. All strains were grown on Mueller‐Hinton broth and agar, and incubated at 37°C for 16 hours unless otherwise is indicated by the supplier. All bacterial strains, NCTC reference numbers and their corresponding resistances are listed in (Table S5). Genomic DNA was prepared from overnight bacterial cultures using the Wizard Genomic DNA Purification Kit (Promega), according to the manufacturer's instructions.

### LAMP assays detection

For fluorescent detection, the 25 μL LAMP reaction mixture contained 15 μL of the isothermal master mix (ISO‐004, Optigene), a final concentration of 0.2 μM of each of the external primers (Integrated DNA technologies), 0.4 μM of each of the loop primers, 1.6 μM of each of the internal primers, 1 μL of DNA template and nuclease‐free water up to the final volume. For colorimetric detection, the 25 μL LAMP reaction mixture contained 12.5 μL of the WarmStart® Colorimetric LAMP 2X master mix (New England Biolabs), with the same concentrations of primers. The LAMP isothermal reaction was performed using a Genie II (Optigene) programmed to incubation at 65°C for 30 min followed by incubation at 98°C for 1 min and a stepwise decrease of 0.05°C/sec to 80°C for the melting temperature analysis.

To assess the LAMP detection limit, a 10‐fold (final DNA quantity of 1000–0.001 pg) and twofold (final DNA quantity of 1–0.0156 pg) serial dilution of *E. coli* NCTC 13846 genomic DNA was used as the DNA template with the LAMP assay for *mcr‐1* as described above.

### Detection of AMR markers from environmental water samples

Tap and pond water samples (150 mL) were collected in separate sterile Duran bottles from a single site in Guildford, Surrey, United Kingdom, during the spring and summer seasons, and transferred to the laboratory before being aliquoted aseptically into sterile 15‐mL tubes. Unspiked water samples were divided into 10 mL aliquots. Spiked water samples were inoculated with bacterial cells (10^7^ cfu/mL), then 10 mL of each water sample was aliquoted into a sterile 15‐mL tube. Water samples were centrifuged at 5000 *g* for 5 min at room temperature. The supernatant was decanted, and the bacterial pellet was resuspended in 100 μL sterile Tris‐EDTA buffer 1X solution (pH 8) (TE) (Fisher BioReagents) and transferred to a 1.5‐mL tube. The bacterial suspension containing 1.5‐mL tubes were then incubated in a heating block at 95°C for 15 min to rupture the bacterial cells before being placed on ice for 5 min. Tubes were then centrifuged at 13,000 *g* for 2 min at room temperature, and 2 μL of the supernatant were used in the LAMP assay as the DNA template, as illustrated in the graphical abstract. For the detection limit, water samples were spiked with *E. coli* NCTC 13846 at a concentration (10^7^–10 cfu/mL). Replicates of spiked water samples were cultured on MacConkey no.3 agar (Oxoid) plates and incubated at 37°C for 24 h to confirm the bacterial count.

Two multiplex real‐time PCR reactions targeting 16S rDNA, *blaKPC*, *blaOXA‐48*, *blaOXA‐23* and *blaVIM* genes were used to validate the LAMP detection and the presence of target AMR markers in tested pond water samples according to (Cahill et al., 2019). Briefly, each 25 μL reaction mixture contained 12.5 μL of the PrimeTime® Gene Expression master mix (Integrated DNA Technologies), a final concentration of 0.5 μM of each of the forward and reverse primers and 0.2 μM probe, 1 μL of DNA template and nuclease‐free water up to the final volume. The first multiplex assay targeted the 16S rDNA (Hex), *blaKPC* (Fam) and *blaOXA‐23* (Cy5), while the second multiplex assay targeted *blaOXA‐48* (Fam) and *blaVIM* (Cy5) genes. The PCR cycles were as follows: 95°C for 10 min and 35 cycles of 95°C for 15 s and 60°C for 1 min and a stepwise increase of 0.5°C for 3 s to 95°C for the melting temperature analysis.

For validation of *mcr‐1* LAMP detection, a standard PCR was performed followed by gel electrophoresis to visualise the amplified 320 bp band according to previously described (Rebelo et al., 2018). Briefly, each 25 μL reaction mixture contained 12.5 μL of the DreamTaq Green PCR Master Mix (ThermoFisher Scientific), a final concentration of 0.2 μM of each of the forward and reverse primers, 1 μL of DNA template and nuclease‐free water up to the final volume. The PCR cycles were as follows: 94°C for 15 min and 25 cycles of 94°C for 30 s, 58°C for 1 min and 72°C for 10 min. The amplicons were visualised by electrophoresis using 1.5% agarose gel containing 1X SYBR Safe (ThermoFisher) at 100 V.

## RESULTS

### Comparative genomics analysis

**FIGURE 1:**
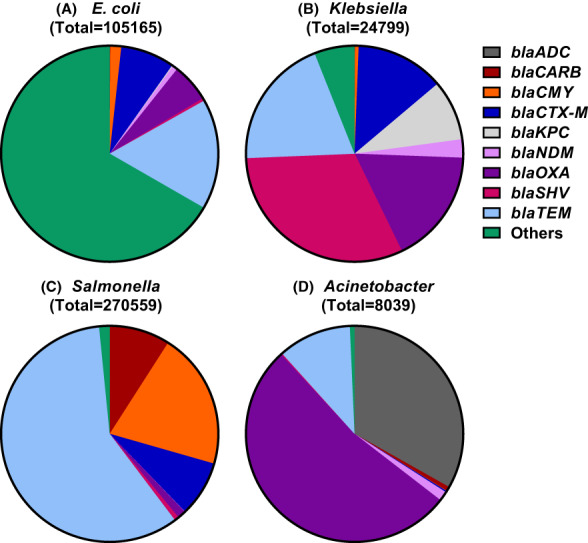
Prevalence of common *bla* genes among beta‐lactamase producing *E. coli*, *Klebsiella*, *Salmonella enterica* and *Acinetobacter* based on enumerated alleles. Enumerated alleles were extracted from the NCBI Pathogens database downloaded datasets between April–May 2020. *blaTEM*, *blaCTX‐M* and *blaOXA* were highly frequent in all four pathogens, while *blaKPC*, *blaCMY* and *blaADC* genes were further incident in *E. coli*, *Klebsiella*, *Salmonella* and *Acinetobacter* genomes, respectively. The comprehensive list of genes included in the others category and the number of alleles of all genes are listed in the (Table S1).

The comparative genomics analysis of *E. coli*, *Klebsiella*, *Salmonella enterica* and *Acinetobacter* revealed the prevalence of beta‐lactamase and *mcr* genes, and gene alleles among the publicly available deposited sequences. The analysis demonstrated that the *E. coli* dataset included 83.9% *E. coli*, 15.8% *Shigella* and 0.3% other species; while the *Klebsiella*, *Salmonella* and *Acinetobacter* datasets had 95.1% *K. pneumoniae*, 99.97% *S. enterica* and 90.7% *A. baumannii*, respectively. The AMR analysis showed that *blaTEM*, *blaCTX‐M*, *blaOXA*, *blaSHV*, *blaCMY*, and *blaKPC* are the most widespread beta‐lactamase genes among all four pathogens (Figure 1, Tables S1 and S2). Figure 1 illustrates the distribution of the most frequent *bla* genes within each of the four pathogens. *Escherichia coli* had high prevalence of *blaTEM*, *blaOXA* and *blaCTX‐M* (Figure 1A), while *blaSHV*, *blaTEM*, *blaOXA*, *blaCTX‐M* and *blaKPC* were highly prevalent in *Klebsiella* (Figure 1B). *Salmonella*, however, presented high prevalence of *blaTEM*, *blaCARB*, *blaCMY*, *blaCTX‐M* and *blaOXA* (Figure 1C). Moreover, *Acinetobacter* demonstrated high prevalence of *blaOXA*, *blaADC* and *blaTEM* (Figure 1D).

**FIGURE 2:**
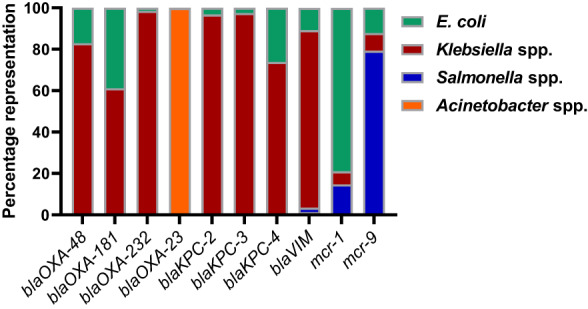
Percentage representation of *blaOXA‐48*, selected *blaOXA‐48‐like* alleles (*blaOXA‐181* and *blaOXA‐232)*, *blaOXA‐23*, *blaKPC* alleles (*blaKPC‐2*, *blaKPC‐3* and *blaKPC‐4*), *blaVIM*, *mcr‐1* and *mcr‐9* genes among *bla* or *mcr* encoding *E. coli*, *Klebsiella*, *Salmonella* and *Acinetobacter* genomes. The percentages were calculated based on the total enumerated counts of *blaOXA‐48* allele (1751), *blaOXA‐181* allele (677), *blaOXA‐232* allele (690), *blaOXA‐23* allele (4068)*, *blaKPC‐2* allele (4896), *blaKPC‐3* allele (2907), *blaKPC‐4* allele (23), *blaVIM* alleles (315), *mcr‐1* allele (1990) and *mcr‐9* alleles (1597), respectively. *Within *blaOXA‐23* alleles, 4063 alleles were for *Acinetobacter* and only three alleles were for *Klebsiella*.

CTX‐M‐15 was the most frequent allele among *blaCTX‐M* enumerated alleles, with CTX‐M alleles showing to be more prevalent in *E. coli* and *Klebsiella* than *Salmonella* and *Acinetobacter*, as predicted (Pitout & Laupland, 2008). CTX‐M‐15 and CTX‐M‐55 were shown to be the most dominant alleles in *Acinetobacter* (Table S3) (Van Vliet et al., 2020). The *blaOXA‐48* and *blaOXA‐48‐like* alleles (*blaOXA‐181* and *blaOXA‐232*) genes were shown to be more widespread in *Klebsiella* followed by *E. coli* (Figure 2). However, the *blaOXA‐23* gene was the most common in *Acinetobacter* and interestingly it has been reported in few isolates of *E. coli* and *Klebsiella* (Figure 2). Moreover, the *blaKPC‐2*, *blaKPC‐3* and *blaKPC‐4* are highly prevalent in *Klebsiella* followed by *E. coli* at different extents, while *blaVIM* gene was most reported with *Klebsiella* followed by *E. coli* and *Salmonella* (Figure 2).

For plasmid‐mediated colistin resistance, *mcr‐1* was highly widespread in *E. coli* followed by *Salmonella* and *Klebsiella*, while mcr‐9 was mainly prevalent in *Salmonella* followed by *E. coli* and *Klebsiella* (Figure 2). *E. coli* also demonstrated high abundance of *mcr‐5*, followed by *mcr‐10*, *mcr‐3* and *mcr‐4* (Table S4). *Klebsiella*, however, demonstrated species‐specific prevalence of 100% of *mcr‐8*, but *mcr‐3*, *mcr‐9* and *mcr‐10* were still detected at less frequent incidence (Table S4). *Salmonella*, nevertheless, presented high incidence of *mcr‐9*, followed by *mcr‐4*, *mcr‐3* and *mcr‐5* (Table S4). *Acinetobacter* demonstrated plasmid‐mediated colistin resistance mainly through acquiring *mcr‐4* (Table S4).

### The development and validation of AMR LAMP assays

**FIGURE 3:**
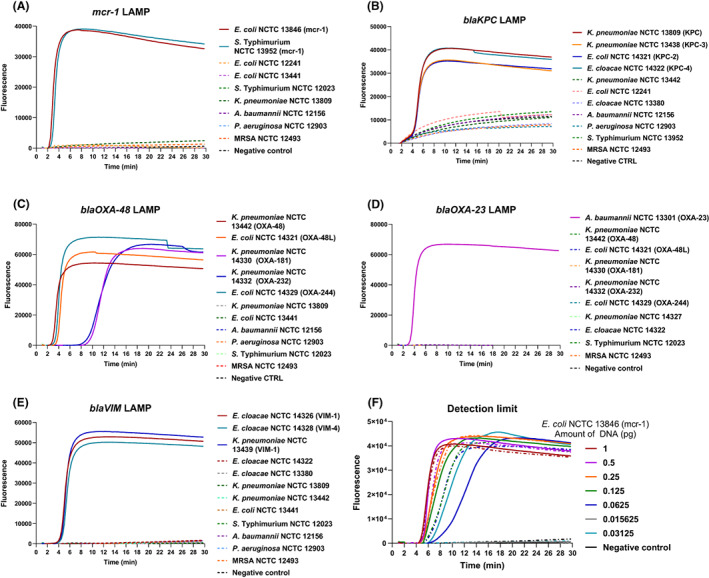
Validation of five AMR LAMP assays. LAMP assays were developed targeting (A). *mcr‐1*, (B) *blaKPC*, (C) *blaOXA‐48*, (D) *blaOXA‐23* and (E) *blaVIM* genes. All assays were validated using strains carrying the resistant targets (solid lines) and negative control strains (dotted lines). All *mcr‐1*, *blaKPC*, *blaOXA‐48*, *blaOXA‐23* and *blaVIM* positive strains showed a successful amplification within less than 10 min. (F) The detection limit of the LAMP assay for *mcr‐1*. 2‐fold serial dilution of 1 pg DNA showing successful amplification of 0.0625 pg DNA (*n* = 2, dotted lines are replicates).

All five AMR LAMP assays were tested and validated against negative control strains that did not have the respective AMR genes, such as *A. baumannii* NCTC 12156, *Pseudomonas aeruginosa* NCTC 12903, *S*. Typhimurium NCTC 12023 and methicillin‐resistant *Staphylococcus aureus* (MRSA) NCTC 12493, to confirm specificity of the assays (Figure 3). The LAMP assay for *mcr‐1* successfully detected *mcr‐1* positive strains *E. coli* NCTC 13846 and *S*. Typhimurium NCTC 13952 within 2 min of amplification, and showed negative results with other tested *E. coli*, *S*. Typhimurium and *K. pneumoniae* strains (Figure 3A). The KPC‐2, KPC‐3 and KPC‐4 alleles were the most frequently detected KPC alleles; therefore, these alleles were targeted for the LAMP assay design and validation. The LAMP assay for *blaKPC* detected all KPC positive strains (*K. pneumoniae* NCTC 13809, *K. pneumoniae* NCTC 13438 (KPC‐3), *E. coli* NCTC 14321 (KPC‐2) and *E. cloacae* NCTC 14322 (KPC‐4)) within 4 min of amplification, while all control strains of *K. pneumoniae*, *E. coli* and *E. cloacae* were negative (Figure 3B). In addition, the *blaOXA‐48* LAMP assay effectively detected OXA‐48 and OXA‐48‐like resistant strains including OXA‐181, OXA‐232 and OXA‐244 alleles within less than 10 min of amplification (Figure 3C). The *blaOXA‐23* LAMP assay also demonstrated detection of OXA‐23 resistant *A. baumannii* strain within 4 min of amplification, while all control strains of *K. pneumoniae*, *E. coli* and *E. cloacae* were negative (Figure 3D). Finally, the *blaVIM* LAMP assay detected VIM‐1 and VIM‐4 alleles, and showed negative results with all tested control strains of *E. cloacae*, *K. pneumoniae* and *E. coli* (Figure 3E). For standardisation, all assays were tested with equal final concentrations of bacterial DNA (4 ng/μL). The specificity of the five developed AMR LAMP assays was thus 100%, and assays showed high sensitivity with overall fluorescent detection within 10 min.

The detection limit of LAMP assay for *mcr‐1* was assessed using a 10‐fold and twofold serial dilutions of genomic DNA of *E. coli* NCTC 13846. The limit of detection was shown to be 0.0625 pg of DNA, which was successfully amplified in duplicates in less than 8 min of the amplification cycle (Figure 3F and S1). The LAMP detection limit was also confirmed through analysing the amplification rate, and confirming the regularity of the amplification peak of all replicates (Figure S1C,D).

### AMR LAMP detection from water samples

**FIGURE 4:**
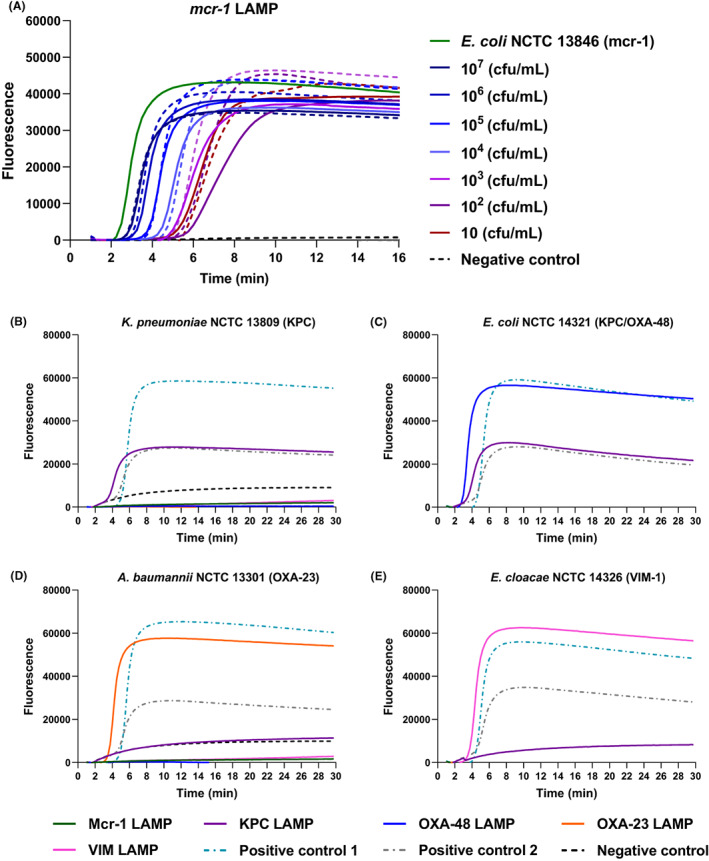
Culture‐independent bacterial detection from spiked tap water samples. (A) Detection limit of the LAMP assay for *mcr‐1* from *E. coli* NCTC 13846 spiked water samples at different spiked bacterial concentrations (*n* = 3). The assay had a detection limit of 10 cfu/mL of spiked bacterial cells in less than 6 min of amplification (solid‐coloured lines, replicates are dotted lines of the same colour). Culture independent LAMP detection of: (B) *blaKPC* (*K. pneumoniae* NCTC 13809), (C) *blaKPC* and *blaOXA‐48* (*E. coli* NCTC 14321), (D) *blaOXA‐23* (*A. baumannii* NCTC 13301) and (E) *blaVIM* (*E. cloacae* NCTC 14326) (solid lines). Positive controls are shown in green and grey dotted lines as follows: B. *blaKPC* LAMP (*K. pneumoniae* NCTC 13438) and *blaVIM* LAMP (*K. pneumoniae* NCTC 13439), C. *blaKPC* LAMP (*K. pneumoniae* NCTC 13438) and *blaOXA‐48* LAMP (*K. pneumoniae* NCTC 13442), D. *blaOXA‐23* LAMP (*A. baumannii* NCTC 13301) and *blaOXA‐48* LAMP (*E. coli* NCTC 14329) and (E) *mcr‐1* LAMP (*S*. Typhimurium NCTC 13952) and *blaVIM* LAMP (*K. pneumoniae* NCTC 13439). Water negative control is represented by a black dotted line, while respective bacterial negative controls are as follows: (B) *blaKPC* LAMP (*E. cloacae* NCTC 14326), (C) *blaOXA‐48* LAMP (*K. pneumoniae* NCTC 13439), (D) *blaKPC* LAMP (*A. baumannii* NCTC 13301) and E. *mcr‐1* LAMP (*E. coli* NCTC 14329).

Tap water samples were collected and spiked with *E. coli* NCTC 13846 (*mcr‐1* positive), at a concentration of 10^7^–10^1^ cfu/mL, to determine the detection limit. The assay had a detection limit of 10 cfu/mL, as confirmed by culture, in less than 6 min of amplification (Figure 4A). To demonstrate simultaneous detection of five AMR targets, bacterial cultures of *K. pneumoniae* NCTC 13809 (*blaKPC* positive), *E. coli* NCTC 14321 (*blaKPC* and *blaOXA‐48* positive), *A. baumannii* NCTC 13301 (*blaOXA‐23* positive) and *E. cloacae* NCTC 14326 (*blaVIM* positive) were individually spiked into tap water, and processed for detection. The *blaKPC* gene was successfully detected with both *K. pneumoniae* NCTC 13809 and *E. coli* NCTC 14321, while *E. coli* NCTC 14321 showed an additional positivity for OXA‐48, as expected (Figure 4B,C). Similarly, OXA‐23 and VIM were detected with *A. baumannii* NCTC 13301 and *E. cloacae* NCTC 14326 spiked samples (Figure 4D,E), respectively.

**FIGURE 5:**
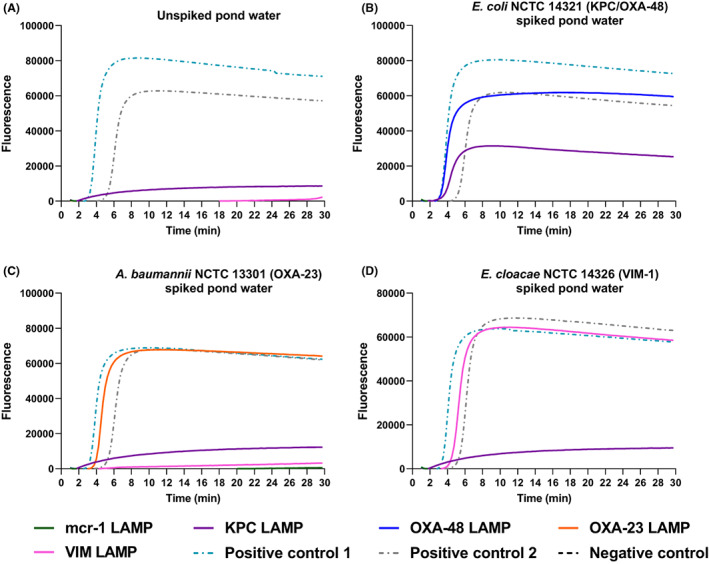
Culture‐independent bacterial detection from pond water samples. (A) Detection from un‐spiked pond water sample. Detection of: (B) *blaKPC* and *blaOXA‐48* (*E. coli* NCTC 14321), (C) *blaOXA‐23* (*A. baumannii* NCTC 13301) and (D) *blaVIM* (*E. cloacae* NCTC 14326) (solid lines) from spiked water samples. Positive controls are shown in green and grey dotted lines as follows: (A) *blaKPC* LAMP (*K. pneumoniae* NCTC 13438) and *mcr‐1* LAMP (*S*. Typhimurium NCTC 13952), (B) *blaOXA‐48* LAMP (*K. pneumoniae* NCTC 13442) and *blaKPC* LAMP (*K. pneumoniae* NCTC 13438), (C) *blaOXA‐23* LAMP (*A. baumannii* NCTC 13301) and *blaOXA‐48* LAMP (*E. coli* NCTC 14329) and (D) *blaVIM* LAMP (*K. pneumoniae* NCTC 13439) and *mcr‐1* LAMP (*E. coli* NCTC 13846). Water negative control is represented by a black dotted line, while respective bacterial negative controls are as follows: (A), (C, D) *blaKPC* LAMP (*E. cloacae* NCTC 14326) and (B) *blaOXA‐48* LAMP (*K. pneumoniae* NCTC 13439).

Similarly, pond water samples were collected and spiked with different bacterial strains for the detection of the five AMR targets. Unspiked pond water samples were negative for all the tested targets, while *E. coli* NCTC 14321 (*blaKPC* and *blaOXA‐48* positive), *A. baumannii* NCTC 13301 (*blaOXA‐23* positive) and *E. cloacae* NCTC 14326 (*blaVIM* positive) spiked pond samples were successfully detected for KPC and OXA‐48, OXA‐23 and VIM, respectively (Figure 5). All results confirmed successful amplification, with no detected inhibition, within 4 min of amplification (Figure 5). The results were confirmed using a validated real‐time PCR assay (Table S6), and showed consistent real‐time PCR detection (Table S7).

**FIGURE 6:**
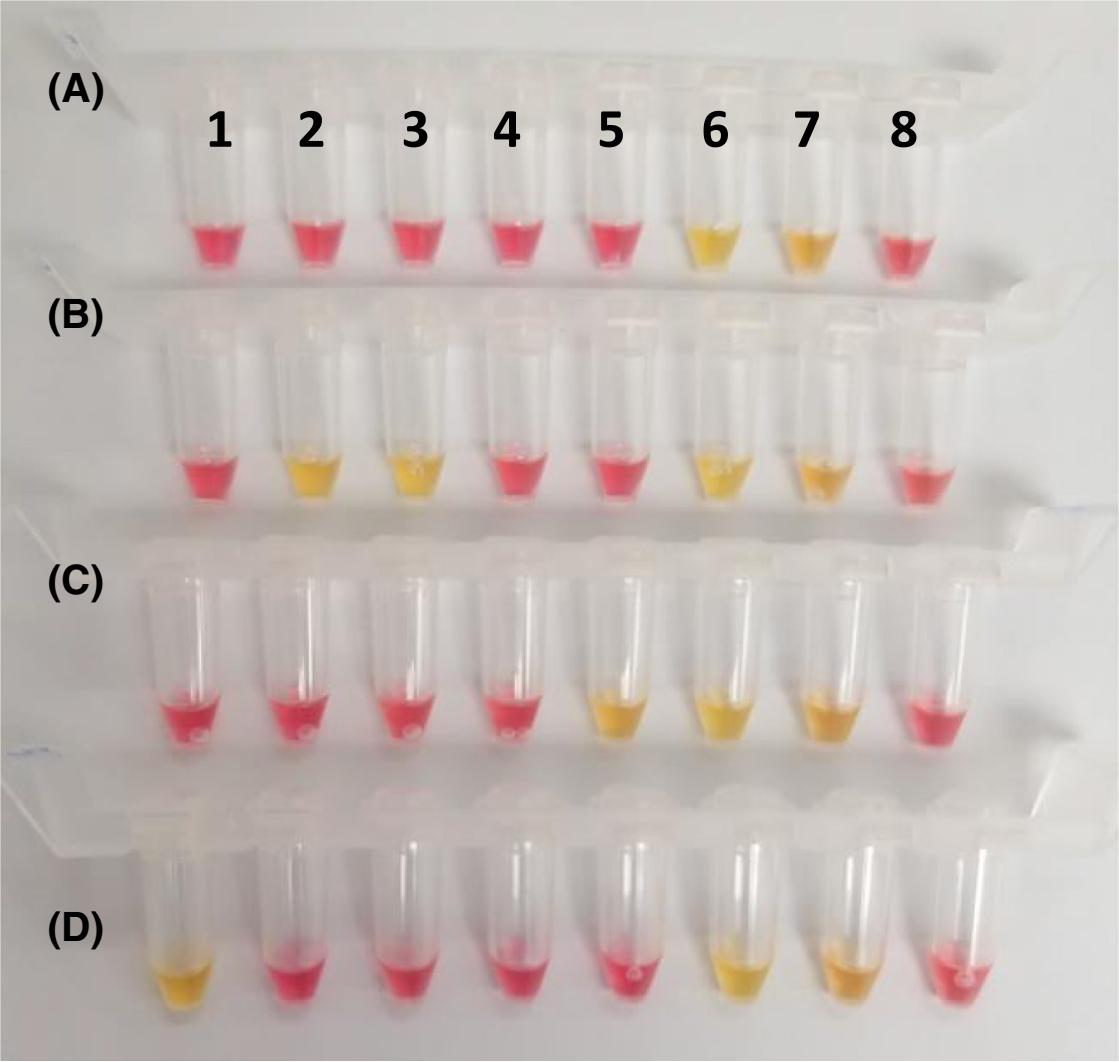
Colorimetric culture‐independent bacterial detection from pond water samples. The 8‐tube strip contains different AMR LAMP primers as follows: (1) *mcr‐1*, (2) *blaKPC*, (3) *blaOXA‐48*, (4) *blaOXA‐23*, (5) *blaVIM*, (6) positive control 1, (7) positive control 2 and (8) negative control. (A) Detection from un‐spiked pond water sample. Detection of: (B) KPC and OXA‐48 (*E. coli* NCTC 14321), (C) VIM (*E. cloacae* NCTC 14326) and (D) mcr‐1 (*S*. Typhimurium NCTC 13952) from spiked pond water samples. Positive controls are as follows: (A) *blaKPC* LAMP (*K. pneumoniae* NCTC 13438) and *mcr‐1* LAMP (*S*. Typhimurium NCTC 13952), (B) *blaOXA‐48* LAMP (*K. pneumoniae* NCTC 13442) and *blaKPC* LAMP (*K. pneumoniae* NCTC 13438), (C) *blaOXA‐23* LAMP (*A. baumannii* NCTC 13301) and *blaVIM* LAMP (*K. pneumoniae* NCTC 13439) and (D) *mcr‐1* LAMP (*E. coli* NCTC 13846) and *blaOXA‐48* LAMP (*E. coli* NCTC 14329). Negative controls are as follows: (A) *blaKPC* LAMP (*A. baumannii* NCTC 13301), (B) *blaOXA‐48* LAMP (*K. pneumoniae* NCTC 13439), (C) *blaVIM* LAMP (*E. coli* NCTC 14329) and (D) *mcr‐1* LAMP (*E. cloacae* NCTC 14326).

To facilitate field‐based detection and detection in resource‐limited settings, we also demonstrated the successful amplification of the developed AMR targets using colorimetric LAMP assays (Figure S2). A colorimetric LAMP assay was also performed using spiked pond water samples to confirm the application of colorimetric detection from environmental water samples. Figure 6 shows the successful culture‐independent detection of the corresponding AMR gene markers (KPC, OXA‐48, VIM and mcr‐1), within less than an hour of sample processing. The results also showed valid positive and negative controls, with no effect on the colour change from red to yellow (Figure 6).

## DISCUSSION

The widespread distribution of beta‐lactamase, especially carbapenemase, is of major concern for human medicine, veterinary medicine and from an environmental perspective. They are often carried on mobile genetic elements facilitating their transfer among bacterial species and increasing their significance in One Health. For instance, *blaKPC* confers carbapenem resistance and is often located as a single copy on conjugative plasmids within a 10 kb mobile transposon (Tn*4401*); however, it can also occur in duplicate (Codjoe & Donkor, 2018; Stoesser et al., 2017). *blaKPC* has now widely spread geographically and has been detected in other bacterial species such as *E. coli*, *E. cloacae* and *Serratia marcescens* (Bryant et al., 2013; Stoesser et al., 2017). Moreover, there are more than 750 types of OXA beta‐lactamases, where OXA‐48 and OXA‐23 belong to class D carbapenemases, due to their ability to rapidly mutate (Codjoe & Donkor, 2018; Pitout et al., 2019). Furthermore, *blaVIM* is of the class B carbapenemases and is often carried on mobile genetic elements that are located within integrons (Codjoe & Donkor, 2018; Sedighi et al., 2014). Additionally, colistin, one of the last resort antibiotics for Gram‐negative bacteria, has been widely misused in food production resulting in an increased plasmid‐mediated colistin resistance (Al‐Tawfiq et al., 2017; Arcilla et al., 2016). *Mcr‐1* has been reported to be carried on multiple plasmids encoding ESBLs in animal and human isolates, which resulted in further widespread of multi‐drug resistances (Arcilla et al., 2016; Wu et al., 2018). Thus, the application of comparative genomics is crucial to identify frequently and newly reported AMR genes and gene alleles and ensure regular monitoring and detection of AMR (Trotter et al., 2019).

In this study, the comparative genomics analysis demonstrated high prevalence of *mcr‐1*, *blaKPC* and *blaOXA* gene markers among publicly available genome sequences of *E. coli*, *Klebsiella*, *Salmonella* and *Acinetobacter*. In *Klebsiella*, the high prevalence of *blaOXA‐48*, *blaOXA‐48‐like*, *blaKPC* and *blaVIM* are concerning, and revealed incidence of these AMR markers among deposited *E. coli* sequences (Figure 2), which can potentially be a source for further AMR dissemination. Similarly, *mcr‐1* was known to be highly prevalent in *E. coli*; however, its current prevalence in *Salmonella* and *Klebsiella* (Figure 2), highlights the need to continuously monitor its dissemination in the environment (Murray et al., 2022). Therefore, these five AMR markers were selected for the development of rapid diagnostics from environmental water samples. It is worth noting that the high occurrence of *blaTEM* among the four pathogens was primarily based on the *blaTEM‐1* allele, which is known to be the most prevalent beta‐lactamase, conferring more than 90% of ampicillin resistance phenotypes to *E. coli* (Ghafourian et al., 2015).

Additionally, we have demonstrated the development and evaluation of five AMR LAMP assays (Table 1 and Figure 3) with 100% sensitivity and specificity against the tested strains, with sensitive detection of 0.0625 pg DNA, in less than 8 min of amplification cycle (Figure 3F and Figure S1). The LAMP detection limit also demonstrated consistency with regard to the amplification peak of all detected replicates through analysing the amplification rate (Figure S1C,D). The developed AMR LAMP assays were aligned with a rapid culture‐independent water sample treatment method. The developed method relies on bacterial concentration, heat‐based cell rupture and precipitation of protein inhibitors to allow for sensitive LAMP detection as illustrated in the graphical abstract. The water sample preparation method was integrated with the validated AMR LAMP assays for culture‐independent bacterial detection from tap water samples at first (Figure 4) before further validations from pond water samples, fluorescently and colorimetric (Figures 5 and 6), respectively. Additionally, the sensitivity of the LAMP detection was validated by qPCR (Tables S6 and S7), which demonstrated successful 16S rRNA and target AMR genes amplification, with early Ct values, of bacterial DNA and pond water samples, respectively. The method is advantageous in offering a rapid, culture‐independent and sensitive detection of 10 cfu/mL of bacterial cells (as confirmed by culture) within 1 h (Figure 4A), which demonstrated an early amplification (<6 min) with minimal inhibition. Furthermore, the method was conducted with a limited use of complicated laboratory procedures and equipment (only requires a centrifuge and a heating block), which are import factors for successful detection at resource‐limited settings.

Contaminated or poor‐quality water represents a great source for the transmission of AMR between animals and humans; however, direct detection often requires filtration and bacterial culture (Hiller et al., 2019). Environmental samples, especially contaminated water, is evidenced to play a role in the evolution and transmission of AMR (Hiller et al., 2019; Huijbers et al., 2015; Larsson & Flach, 2022). Therefore, the water sample processing method was developed and optimised to rapidly detect AMR genes without the need for culture‐based methods. The integrated LAMP AMR detection from environmental water samples will allow the screening of AMR markers especially in outbreaks, limited‐resource settings and/or low‐ and middle‐income countries to enhance AMR surveillances (Trotter et al., 2019). The current process can be further extended for the detection of other important AMR targets and gene alleles for the surveillance of AMR in environmental water samples and irrigation water. It can be further implemented in wastewater‐based surveillances, as the current SARS‐CoV‐2 pandemic demonstrated the applicability of wastewater‐based monitoring of infectious diseases (Hillary et al., 2021).

## CONCLUSION

There is an urgent need for rapid and economic diagnostics for the detection of bacterial pathogens and AMR in environmental water samples that can be easily deployed in remote settings. However, the current standard methods rely on culturing steps, which affects sensitivity and are time‐consuming. Here, we demonstrated the development of a rapid and sensitive water sample preparation method integrated with the detection of five AMR markers using a LAMP‐based assay. The LAMP assays were thoroughly evaluated, detected target genes within less than 10 min, and showed 100% sensitivity and specificity with a detection limit of 0.0625 pg DNA. The study demonstrated the culture‐independent detection of *mcr‐1*, *blaKPC*, *blaOXA‐48*, *blaOXA‐23* and *blaVIM* from tap and pond water using fluorescent and colorimetric detection, for ease of data interpretation. The integrated AMR LAMP detection from water samples demonstrated low‐level detection of 10 cfu/mL bacterial cells. The AMR water LAMP detection technique developed here will facilitate the rapid and sensitive detection of AMR in remote locations and low‐ and middle‐income countries, which will help increase the surveillance of AMR data and reduce associated health and economic burdens.

## AUTHOR CONTRIBUTIONS

**Marwa M. Hassan:** Conceptualization (lead); data curation (lead); investigation (lead); methodology (lead); project administration (supporting); writing – original draft (lead); writing – review and editing (lead). **Arnoud H. M. van Vliet:** Conceptualization (equal); data curation (equal); funding acquisition (supporting); investigation (equal); methodology (equal); project administration (lead); writing – review and editing (equal). **Owen Higgins:** Conceptualization (supporting); investigation (supporting); methodology (supporting); project administration (supporting); writing – review and editing (supporting). **Liam P. Burke:** Conceptualization (supporting); investigation (supporting); methodology (supporting); project administration (equal); writing – review and editing (supporting). **Alexandra Chueiri:** Conceptualization (supporting); investigation (supporting); methodology (supporting); project administration (supporting); writing – review and editing (supporting). **Louise O'Connor:** Conceptualization (supporting); investigation (supporting); methodology (equal); project administration (supporting); writing – review and editing (supporting). **Dearbháile Morris:** Conceptualization (supporting); investigation (supporting); methodology (supporting); project administration (supporting); writing – review and editing (supporting). **Terry J. Smith:** Conceptualization (supporting); funding acquisition (lead); investigation (supporting); methodology (supporting); project administration (lead); writing – review and editing (supporting). **Roberto La Ragione:** Conceptualization (equal); funding acquisition (lead); investigation (supporting); methodology (supporting); project administration (lead); writing – review and editing (equal).

## CONFLICT OF INTEREST STATEMENT

The authors have no conflict of interest.

## Supporting information


**Appendix S1.** **Supporting Information.**
