# Supplementary material for: Rapid culture‐independent loop‐mediated isothermal amplification detection of antimicrobial resistance markers from environmental water samples
Source: Microb Biotechnol. 2023 Feb 3;16(5):977–89. doi: 10.1111/1751-7915.14227 (PMC10128135; doi:10.1111/1751-7915.14227)
Supplement: Supplementary file 1 — Appendix S1. Supporting Information. [file MBT2-16-977-s001.docx]

**Supplementary information for**

**Rapid culture-independent loop-mediated isothermal amplification (LAMP) detection of antimicrobial resistance markers from environmental water samples**

Marwa M. Hassan^1^, Arnoud H. M. van Vliet^1^, Owen Higgins^2^, Liam P. Burke^3, 4^, Alexandra Chueiri^3, 4^, Louise O'Connor^3, 4^, Dearbháile Morris^3, 4^, Terry Smith^2^ and Roberto M. La Ragione^1, 5*^

^1^Department of Comparative Biomedical Sciences, School of Veterinary Medicine, Faculty of Health and Medical Sciences, University of Surrey, Guildford, Surrey, United Kingdom.

^2^Molecular Diagnostics Research Group, School of Biological and Chemical Sciences, University of Galway, Ireland.

^3^Antimicrobial Resistance and Microbial Ecology Group, School of Medicine, University of Galway, Ireland.

^4^Centre for One Health, Ryan Institute, University of Galway, Ireland.

^5^School of Biosciences, Faculty of Health and Medical Sciences, University of Surrey, Guildford, Surrey, United Kingdom.

***Corresponding authors:** [r.laragione@surrey.ac.uk](mailto:r.laragione@surrey.ac.uk); m.hussainalihassan@surrey.ac.uk

**Figure S1. The detection limit of the *mcr-1* LAMP assay. A.** 10-fold serial dilution of 1000 pg DNA showing successful amplification of 0.1 pg DNA (n=2). **B.** 2-fold serial dilution of 1 pg DNA showing successful amplification of 0.0625 pg DNA (n=2). **C.** and **D.** The amplification rate for both assays demonstrated a consistent result.

**
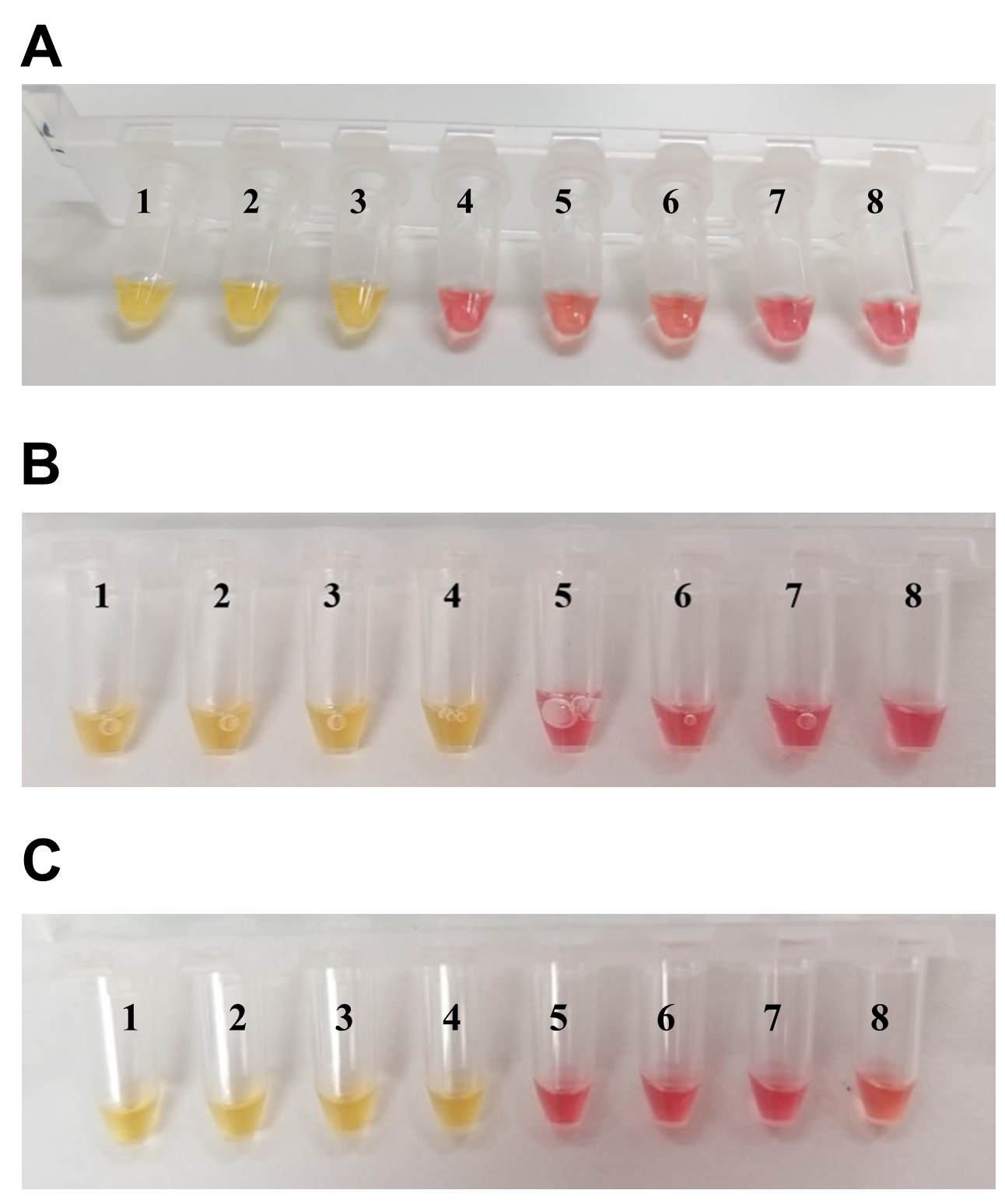
**

**Figure S2. The colorimetric *mcr-1* and KPC LAMP assays and detection limit. A. The *mcr-1* LAMP** **assay.** Tubes from left to right: **1.** *E. coli* NCTC 13846 (*mcr-1*), **2.** *S.* Typhimurium NCTC 13952 (*mcr-1*), **3.** *E. coli* A9 pJMA (*mcr-1*), **4.** *E. coli* NCTC 12241, **5.** *S.* Typhimurium NCTC 12023, **6.** *A. baumannii* NCTC 12156, **7.** *K. pneumoniae* NCTC 13809 and **8.** Negative control. **B. KPC LAMP** **assay.** Tubes from left to right: **1.** *K. pneumoniae* NCTC 13809 (*blaKPC*)**, 2.** *K. pneumoniae* NCTC 14327 (*blaKPC*)**, 3.** *E. coli 14321* NCTC (*blaKPC*)**, 4.** *E. cloacae* NCTC 14322 (*blaKPC*)**, 5.** *K. pneumoniae* NCTC 13439**, 6.** *E. cloacae* NCTC *13380***, 7.** *E. coli* NCTC 13441 and **8.** Negative control. **C.** **The limit of detection of mcr-1 LAMP assay using *E. coli* NCTC 13846 DNA.** A 10-fold serial dilution of **1.** 10000, **2.** 1000, **3.** 100, **4.** 10, **5.** 1, **6.** 0.1, **7.** 0.01 pg DNA and **8.** Negative control.

**Table S1. The total enumerated counts of detected beta-lactamase alleles among *E. coli*, *Klebsiella*, *Salmonella* *enterica* and *Acinetobacter* genome sequences.**

| *bla* (all) | *E. coli* (n=105165) | *Klebsiella* (n=24799) | *Salmonella* (n=270559) | *Acinetobacter* (n=8039) |
| --- | --- | --- | --- | --- |
| *blaACC** | 10 | 8 | 11 | 0 |
| *blaACT** | 7 | 5 | 2 | 0 |
| *blaADC* | 0 | 4 | 0 | 8181 |
| *blaBEL** | 0 | 5 | 0 | 0 |
| *blaBPU** | 0 | 0 | 1 | 0 |
| *blaCARB* | 149 | 46 | 4851 | 157 |
| *blaCMH** | 0 | 1 | 0 | 0 |
| *blaCMY* | 2574 | 471 | 10956 | 13 |
| *blaCTX-M* | 12696 | 11301 | 4487 | 52 |
| *blaDHA** | 265 | 639 | 124 | 4 |
| *blaEC** | 104330 | 11 | 26 | 4 |
| *blaFOX** | 6 | 31 | 3 | 0 |
| *blaFRI** | 3 | 9 | 0 | 0 |
| *blaGES** | 21 | 40 | 0 | 27 |
| *blaGIL** | 1 | 0 | 0 | 0 |
| *blaHER** | 310 | 0 | 248 | 0 |
| *blaI** | 89 | 193 | 2 | 42 |
| *blaIMI** | 1 | 2 | 0 | 0 |
| *blaIMP** | 86 | 189 | 0 | 41 |
| *blaKLUC** | 0 | 2 | 2 | 0 |
| *blaKPC* | 246 | 7737 | 3 | 2 |
| *blaLAP** | 89 | 1137 | 231 | 0 |
| *blaLAT** | 1 | 0 | 0 | 0 |
| *blaLEN** | 1 | 813 | 0 | 0 |
| *blaMOX** | 1 | 4 | 0 | 0 |
| *blaNDM* | 1175 | 2350 | 15 | 345 |
| *blaOKP** | 2 | 949 | 0 | 0 |
| *blaOXA* | 9129 | 14757 | 665 | 12986 |
| *blaOXY** | 0 | 417 | 0 | 0 |
| *blaPC** | 1 | 0 | 0 | 0 |
| *blaPSE** | 12 | 5 | 39 | 15 |
| *blaROB** | 1 | 0 | 0 | 0 |
| *blaSCO** | 10 | 289 | 140 | 0 |
| *blaSFO** | 3 | 14 | 0 | 0 |
| *blaSHV* | 602 | 26989 | 358 | 42 |
| *blaSRT** | 3 | 0 | 0 | 0 |
| *blaTEM* | 26100 | 16819 | 31498 | 2696 |
| *blaVEB** | 9 | 101 | 6 | 33 |
| *blaVIM** | 34 | 270 | 7 | 4 |
| *blaZ** | 4 | 2 | 0 | 1 |

* These were enumerated as ‘Others’ in Figure 1.

**Table S2. The percentage of detected beta-lactamase alleles among *E. coli*, *Klebsiella*, *Salmonella* *enterica* and *Acinetobacter* genome sequences.** Percentages were calculated as a representation among the four detected pathogens. Grey highlighted rows are represented in Figure 1.

| *bla* (all) | The percentage in | | | |
| --- | --- | --- | --- | --- |
|  | *E. coli* | *Klebsiella* | *Salmonella* | *Acinetobacter* |
| *blaACC** | 34.48 | 27.59 | 37.93 | 0.00 |
| *blaACT** | 50.00 | 35.71 | 14.29 | 0.00 |
| *blaADC* | 0.00 | 0.05 | 0.00 | 99.95 |
| *blaBEL** | 0.00 | 100.00 | 0.00 | 0.00 |
| *blaBPU** | 0.00 | 0.00 | 100.00 | 0.00 |
| *blaCARB* | 2.86 | 0.88 | 93.23 | 3.02 |
| *blaCMH** | 0.00 | 100.00 | 0.00 | 0.00 |
| *blaCMY* | 18.37 | 3.36 | 78.18 | 0.09 |
| *blaCTX-M* | 44.49 | 39.60 | 15.72 | 0.18 |
| *blaDHA** | 25.68 | 61.92 | 12.02 | 0.39 |
| *blaEC** | 99.96 | 0.01 | 0.02 | 0.00 |
| *blaFOX** | 15.00 | 77.50 | 7.50 | 0.00 |
| *blaFRI** | 25.00 | 75.00 | 0.00 | 0.00 |
| *blaGES** | 23.86 | 45.45 | 0.00 | 30.68 |
| *blaGIL** | 100.00 | 0.00 | 0.00 | 0.00 |
| *blaHER** | 55.56 | 0.00 | 44.44 | 0.00 |
| *blaI** | 27.30 | 59.20 | 0.61 | 12.88 |
| *blaIMI** | 33.33 | 66.67 | 0.00 | 0.00 |
| *blaIMP** | 27.22 | 59.81 | 0.00 | 12.97 |
| *blaKLUC** | 0.00 | 50.00 | 50.00 | 0.00 |
| *blaKPC* | 3.08 | 96.86 | 0.04 | 0.03 |
| *blaLAP** | 6.11 | 78.04 | 15.85 | 0.00 |
| *blaLAT** | 100.00 | 0.00 | 0.00 | 0.00 |
| *blaLEN** | 0.12 | 99.88 | 0.00 | 0.00 |
| *blaMOX** | 20.00 | 80.00 | 0.00 | 0.00 |
| *blaNDM* | 30.24 | 60.49 | 0.39 | 8.88 |
| *blaOKP** | 0.21 | 99.79 | 0.00 | 0.00 |
| *blaOXA* | 24.32 | 39.31 | 1.77 | 34.60 |
| *blaOXY** | 0.00 | 100.00 | 0.00 | 0.00 |
| *blaPC** | 100.00 | 0.00 | 0.00 | 0.00 |
| *blaPSE** | 16.90 | 7.04 | 54.93 | 21.13 |
| *blaROB** | 100.00 | 0.00 | 0.00 | 0.00 |
| *blaSCO** | 2.28 | 65.83 | 31.89 | 0.00 |
| *blaSFO** | 17.65 | 82.35 | 0.00 | 0.00 |
| *blaSHV* | 2.15 | 96.42 | 1.28 | 0.15 |
| *blaSRT** | 100.00 | 0.00 | 0.00 | 0.00 |
| *blaTEM* | 33.85 | 21.81 | 40.85 | 3.50 |
| *blaVEB** | 6.04 | 67.79 | 4.03 | 22.15 |
| *blaVIM** | 10.79 | 85.71 | 2.22 | 1.27 |
| *blaZ** | 57.14 | 28.57 | 0.00 | 14.29 |

* These were enumerated as ‘Others’ in Figure 1.

**Table S3. The prevalence of *blaCTX-M* alleles among *E. coli*, *Klebsiella*, *Salmonella enterica* and *Acinetobacter* genome sequences.** The percentages were calculated based on the total enumerated alleles of the four pathogens as 100% to demonstrate pathogen-specific prevalences.

| *blaCTX-M* (allele counts) | The percentage in | | | |
| --- | --- | --- | --- | --- |
|  | ***E. coli*** | ***Klebsiella*** | ***Salmonella*** | ***Acinetobacter*** |
| *blaCTX-M** | 44.49 | 39.60 | 15.72 | 0.18 |
| *blaCTX-M-1* (1147)** | 85.61 | 3.75 | 10.64 | 0.00 |
| *blaCTX-M-14* (2357)** | 66.65 | 28.43 | 4.92 | 0.00 |
| *blaCTX-M-15* (14377)** | 42.08 | 55.29 | 2.50 | 0.13 |
| *blaCTX-M-27* (1591)** | 90.01 | 9.05 | 0.75 | 0.19 |
| *blaCTX-M-55* (1372)** | 71.94 | 8.53 | 18.59 | 0.95 |
| *blaCTX-M-65* (4196)** | 5.93 | 23.02 | 71.02 | 0.02 |

*The percentages were calculated based on all enumerated *bla* genes with a total of 28536 alleles for *blaCTX-M*. **The percentages were calculated based on the total enumerated counts of *blaCTX-M-1*, *blaCTX-M-14*, *blaCTX-M-15*, *blaCTX-M-27*, *blaCTX-M-55 and blaCTX-M-65* alleles, respectively.

**Table S4. The prevalence of detected *mcr* genes among *E. coli*, *Klebsiella*, *Salmonella enterica* and *Acinetobacter* genome sequences.** The percentages were calculated based on the enumerated counts for each allele within the total enumerated counts for the four pathogens to demonstrate pathogen-specific prevalences.

| *mcr* genes (allele counts) | The percentage in | | | |
| --- | --- | --- | --- | --- |
|  | ***E. coli*** | ***Klebsiella*** | ***Salmonella*** | ***Acinetobacter*** |
| *mcr-1* (1990) | 78.94 | 6.38 | 14.57 | 0.10 |
| *mcr-3* (189) | 57.14 | 12.70 | 30.16 | 0.00 |
| *mcr-4* (25) | 20.00 | 0.00 | 60.00 | 20.00 |
| *mcr-5* (78) | 71.79 | 0.00 | 28.21 | 0.00 |
| *mcr-8* (50) | 0.00 | 100.00 | 0.00 | 0.00 |
| *mcr-9* (1597) | 12.21 | 8.52 | 79.27 | 0.00 |
| *mcr-10* (17) | 58.82 | 41.18 | 0.00 | 0.00 |

**Table S5. Bacterial strains used in this study and encoded resistant genes.**

| Bacterial strain | Reference numbers | Encoded resistance |
| --- | --- | --- |
| *E. coli* | NCTC 12241 | Control strain |
|  | NCTC 13846 | *mcr-1* |
|  | NCTC 14321 | *blaKPC-2* and *blaOXA-48-like* |
|  | NCTC 13441 | *blaCTX-M-15* |
|  | NCTC 14329 | *blaOXA-244* (OXA-48-like) |
| *S.* Typhimurium | NCTC 13952 | *mcr-1* |
|  | NCTC 12023 | Control strain |
| *K. pneumoniae* | NCTC 13809 | *blaKPC* |
|  | NCTC 13438 | *blaKPC-3* |
|  | NCTC 13442 | *blaOXA-48* |
|  | NCTC 14330 | *blaOXA-181* (OXA-48-like) |
|  | NCTC 14332 | *blaOXA-232* (OXA-48-like) |
|  | NCTC 14327 | *blaKPC* |
|  | NCTC 13439 | *blaVIM-1* |
| *E. cloacae* | NCTC 13380 | cephalosporinase beta-lactamase II |
|  | NCTC 14322 | *blaKPC-4* |
|  | NCTC 14326 | *blaVIM-1* |
|  | NCTC 14328 | *blaVIM-4* |
| *A. baumannii* | NCTC 12156 | Control strain |
|  | NCTC 13301 | *blaOXA-23* and *blaOXA-51-like* |
| *P. aeruginosa* | NCTC 12903 | Control strain |
| MRSA | NCTC 12493 | *mec-A* |

**Table S6. Real-time PCR validation Ct values for target AMR genes using bacterial DNA**. (Mean Ct ± SD (n=3), N.D. not detected)**.**

| Target | | *E. coli* NCTC 14321 (KPC/OXA-48) | *A. baumannii* NCTC 13301 (OXA-23) | *E. cloacae* NCTC 14326 (VIM) | *E. coli* NCTC 12241 | Negative control |
| --- | --- | --- | --- | --- | --- | --- |
| **Assay 1** | **KPC** | 17.94 ± 0.02 | N.D. | N.D. | N.D. | N.D. |
|  | **OXA-23** | N.D. | 18.14 ± 0.26 | N.D. | N.D. | N.D. |
|  | **16S rDNA** | 16.13 ± 0.07 | 16.29 ± 0.32 | 14.31 ± 0.13 | 15.87 ± 0.14 | N.D. |
| **Assay 2** | **OXA-48** | 19.31 ± 0.06 | N.D. | N.D. | N.D. | N.D. |
|  | **VIM** | N.D. | N.D. | 17.58 ± 0.18 | N.D. | N.D. |

**Table S7. Real-time PCR detection Ct values for target AMR genes from un-spiked and bacterial spiked pond water samples**. (Mean Ct ± SD (n=3), N.D. not detected)**.**

| Target | | Un-spiked sample 1 | Un-spiked sample 2 | *E. coli* NCTC 14321 (KPC/OXA-48) | *A. baumannii* NCTC 13301 (OXA-23) | *E. cloacae* NCTC 14326 (VIM) |
| --- | --- | --- | --- | --- | --- | --- |
| **Assay 1** | **KPC** | N.D. | N.D. | 18.82 ± 0.27 | N.D. | N.D. |
|  | **OXA-23** | N.D. | N.D. | N.D. | 23.39 ± 0.22 | N.D. |
|  | **16S rDNA** | 25.38 ± 0.22 | 28.11 ± 0.17 | 17.34 ± 0.25 | 21.37 ± 0.29 | 17.48 ± 0.06 |
| **Assay 2** | **OXA-48** | N.D. | N.D. | 20.00 ± 0.14 | N.D. | N.D. |
|  | **VIM** | N.D. | N.D. | N.D. | N.D. | 19.77 ± 0.16 |
